# First report of a bla_NDM_-resistant gene in a *Klebsiella aerogenes* clinical isolate from Brazil

**DOI:** 10.1590/0037-8682-0262-2020

**Published:** 2020-12-21

**Authors:** Cynthia Regina Pedrosa Soares, Jorge Belém Oliveira-Júnior, Elza Ferreira Firmo

**Affiliations:** 1 Universidade Federal de Pernambuco, Departamento de Medicina Tropical, Recife, PE, Brasil.; 2 Instituto Aggeu Magalhães, Laboratório de Biologia Molecular e Celular, Departamento de Parasitologia, Recife, PE, Brasil.

**Keywords:** Klebsiella aerogenes, *bla*_NDM_ gene, metallo-betalactamase, Carbapenemase, Multidrug-resistant

## Abstract

**INTRODUCTION:**

Carbapenemase-resistant enterobacteria that produce the *bla*_NDM_ gene are found worldwide. However, this is the first report of bla_NDM_ in *Klebsiella aerogenes* in Brazil.

**METHODS:**

The identification of bacterial species was performed using anautomated system and confirmed by biochemical tests, 16S rRNA gene sequencing, and detection of resistance genes.

**RESULTS:**

The clinical isolate showed minimum inhibitory concentration resistance to meropenem and polymyxin B at 8mg/L and 4mg/L, respectively. Only the bla_NDM_ gene was detected.

**CONCLUSIONS:**

The current report of the bla_NDM_ gene in isolated MDR enterobacteria indicates that this gene can spread silently in a hospital setting.

New Delhi metallo-beta-lactamase (NDM), which codes for the *bla*_NDM_ gene, is found in carbapenemase-producing Enterobacteriaceae, and the highest distributions of *bla*_NDM_ variants have been detected in *Klebsiella pneumoniae* and *Escherichia coli* ^1^ ^,^ ^2^. The occurrence of the *bla*_NDM_ gene in *Klebsiella aerogenes* has also been documented in countries such as China^3^, South Korea^4^, Tunisia^5^, Japan^6^, and India^7^ ^,^ ^8^. In South America, the *bla*_NDM_ gene was first reported in 2012 in Uruguay^9^. In Brazil, this gene has been detected in only 11 isolates (0.97%) from Porto Alegre, with nine from *Enterobacter cloacae* and two from *Morganella morganii* complexes. Moreover, other isolates showed high resistance to carbapenems^10^. The spread of the *bla*_NDM_ gene in various species of gram-negative bacilli has occurred in different Brazilian regions^11^. In 2019, the first report of *Proteus mirabilis* and *Serratia marcescens* carrying *bla*_KPC-2_ and *bla*_NDM-1_ genes in Brazil was published^12^. The *bla*_NDM-1_ gene product is NDM-1, which hydrolyzes a wide range of β-lactam antimicrobials, including carbapenems, which are the last-resort antimicrobials in the treatment of infections caused by antimicrobial-resistant bacteria. The emergence of bacteria carrying these genes represents a challenge in the treatment of infections^1^.The objective of this study was to describe the occurrence of the *bla*_NDM_ gene in a clinical isolate of *K. aerogenes* resistant to aminopenicillin, beta-lactam + beta-lactamase inhibitors, and 1st, 2nd, 3rd, and 4th generation beta-lactams, as well as carbapenems.

Identification of bacterial species was performed usinga Vitek-2 automated system (bioMérieux, Marcy I’Etoile, France), and confirmed by biochemical tests and 16S rRNA gene sequencing. PCR and DNA sequencing were performed to track the presence of the *bla*_NDM_ gene in two duplicates, collected at a referral hospital in Recife (Pernambuco, Brazil) in 2019. A universal primer pair, NDM-F (5-CGGAATGGCTCATCACGATC-3) and NDM-R (5-GGTTTGGCGATCTGGTTTTC-3)^13^were used for screening, as previously described. Furthermore, the entire *bla*_NDM_ region was amplified and sequenced based on consensus sequences flanking the gene. The analysis showed that the *bla*_NDM_ gene was present in a *K. aerogenes* clinical isolate from a patient with bacteremia hospitalized in an intensive care unit (ICU). The minimum inhibitory concentrations (MIC) of meropenem and polymyxin B for this isolate were 8mg/L and 4mg/L, respectively. In addition, this isolate presented a susceptibility of 8mg/L to amikacin and 1mg/L to colistin, according to the Brazilian Committee on Antimicrobial Susceptibility Testing (BrCAST)^14^. These data were confirmed by colorimetric assays with resazurin^15^.

The *K. aerogenes* isolate showed an antimicrobial resistance profile (R) to cefepime, ceftazidime, cefoxitin, ceftriaxone, ciprofloxacin, ampicillin, ampicillin/sulbactam, cefotaxime, and carbapenems (imepenem, meropenem, ertapenem), which differs from its resistance profile in China^3^. High resistance to carbapenems has also been observed in other Enterobacteriaceae isolates^10^. However, it was susceptible (S) to amikacin, gentamicin, tobramycin, tigecycline, colistin, and polymyxin B, and sensitive, with increasing exposure (I), to ciprofloxacin, according to BrCAST^14^. The present study detected the presence of the broad-spectrum beta-lactamase and beta-lactamase class C beta-lactamase phenotypes in *K. aerogenes* isolates. The presence of *bla*_NDM_ was confirmed by polymerase chain reaction (PCR). To our knowledge, this is the first report of the *bla*_NDM_ gene in clinical isolates of *K. aerogenes* from Brazil. In addition, other carbapenemase genes, such as *bla*_IMP_, *bla*_VIM_, *bla*_GES_, *bla*_OXA-48_, and *bla*_KPC_ were investigated, as well as the mcr-1 gene. Despite being susceptible to polymyxin B by automated testing, the MIC showed resistance, but the mcr-1 gene was not detected. However, none of the other genes were detected in the clinical isolates used. NDM-producing strains, including carbapenems, cephamycin, extended-spectrum cephalosporins, aminoglycosides, monobactamines, tetracyclines, fluoroquinolones, and polymyxin and colistin^8^, were detected in neonates in the ICU with high antibiotic resistance. 

NDM-1-producing Enterobacteriaceae has spread rapidly in hospitalized patients in several countries. Thus, the spread of carbapenemase-resistant genes has been a major concern worldwide, as it poses a threat to the antimicrobial treatment of infections caused by multidrug-resistant isolates. Although strains that produce *bla*_NDM_ appear less frequently in Brazil, it is nonetheless necessary to control epidemiological surveillance to control infections. 

**Nucleotide sequence accession number:** The nucleotide sequence of the bla_NDM_ gene in the *K. aerogenes* isolate was GenBank/EMBL/DDBJ, accession Nº. MN735678.1. 

This study was approved by the ethics committee of our institution (CAAE: 20195119.3.0000.5586), opinion number 3,787,364, and (CAAE: 20195119.3.3001.5198) opinion number 3,838,892, following Resolution 466/12 of the National Health Council.
